# The Q2 Mitochondrial Haplogroup in Oceania

**DOI:** 10.1371/journal.pone.0052022

**Published:** 2012-12-20

**Authors:** Chris A. Corser, Patricia A. McLenachan, Melanie J. Pierson, G. L. Abby Harrison, David Penny

**Affiliations:** 1 Institute of Molecular BioSciences, Massey University, Palmerston North, New Zealand; 2 Department of Anthropology, University of Auckland, Auckland, New Zealand; 3 Peter Medawar Building for Pathogen Research, Oxford University, Oxford, United Kingdom; Erasmus University Medical Center, The Netherlands

## Abstract

Many details surrounding the origins of the peoples of Oceania remain to be resolved, and as a step towards this we report seven new complete mitochondrial genomes from the Q2a haplogroup, from Papua New Guinea, Fiji and Kiribati. This brings the total to eleven Q2 genomes now available. The Q haplogroup (that includes Q2) is an old and diverse lineage in Near Oceania, and is reasonably common; within our sample set of 430, 97 are of the Q haplogroup. However, only 8 are Q2, and we report 7 here. The tree with all complete Q genomes is proven to be minimal. The dating estimate for the origin of Q2 (around 35 Kya) reinforces the understanding that humans have been in Near Oceania for tens of thousands of years; nevertheless the Polynesian maternal haplogroups remain distinctive. A major focus now, with regard to Polynesian ancestry, is to address the differences and timing of the ‘Melanesian’ contribution to the maternal and paternal lineages as people moved further and further into Remote Oceania. Input from other fields such as anthropology, history and linguistics is required for a better understanding and interpretation of the genetic data.

## Introduction

Oceania was historically divided into: Melanesia (including Papua New Guinea, the Solomon Islands, New Caledonia, Fiji and Vanuatu); Micronesia (including the Mariana Islands, the Federated States of Micronesia, the Marshall Islands, Kiribati); and Polynesia (including Tonga, Samoa, the Cook Islands, Hawaii, French Polynesia, New Zealand). For human prehistory, it is more naturally divided into Near and Remote Oceania with the boundaries of Near Oceania encompassing Papua New Guinea and the Solomon Islands, and Remote Oceania the other more eastern islands in the Pacific. The most commonly accepted general model for the peopling (settlement) of Oceania is that there were two main periods of migration into the region. Firstly, there was an early settlement (starting 40–50,000 years ago into Australia, New Guinea, Near Oceania), and then a more recent (4–5,000 years BP) intrusion into Near Oceania of Austronesian-speaking peoples followed eventually by the relatively rapid settlement of the many islands of Remote Oceania [1], [2], [3].

Although many aspects of the human settlement of Polynesia have been resolved, more information is still being amassed from a wide range of disciplines including archaeology [1], linguistics [4]–[6] anthropology [7], technology [8] and infectious diseases [9], [10] to help decipher the origins of Pacific populations. Currently a spectrum of models exists, ranging from the ‘entangled bank’ to the ‘express train’, that aim to explain the prehistoric migration patterns of the Polynesian peoples [2]. The complexity of social data in the region [11] implies that in general, most models have several components. It is important to fully specify the models and which components are being tested - for example, there are at least seven independent components to the ‘express train’ model including three related to Taiwan (language, associated culture, and the maternal genetics from mitochondrial DNA), three related to the rate of movement (including a relatively rapid movement, no significant breaks after leaving Taiwan until reaching Western Polynesia, little displacement of existing peoples), and finally the question of ongoing genetic contact with other peoples in the region – including differences between introgression of males and females [12]. Also, different models can have the same name; an example being the ‘slow boat’ models of Kayser et al. [13] and of Oppenheimer and Richards [14]. While we do not attempt to formally evaluate the main models here (but see Hurles et al, 2003 [2]), it is important to be aware that most models have several compon that can be evaluated independently, and by using data from a range of disciplines.

In some cases in Polynesia it has been possible to estimate founding population sizes [15]–[17]. However, for the broader region we require considerably more data on both maternal and paternal genetic diversity before we can begin to model the founding populations. The immediate question is whether the mitochondrial data fit the two stage model of migration into Near Oceania (an early occupation and a later Austronesian intrusion). And then if so, does this model require a very large initial founding population to explain the high mitochondrial diversity in the region? Or is there also an intermediate intrusion of peoples from SE Asia, as may occur in eastern Indonesia [18]? Or did this diversity, such as the Q, and then the Q2 haplogroup, arise later within Near Oceania? In addition, to better understand the origins of the people of Polynesia it is necessary to more fully elucidate the potential Near Oceania influence. Data from this study contributes to these questions.

**Figure 1:**
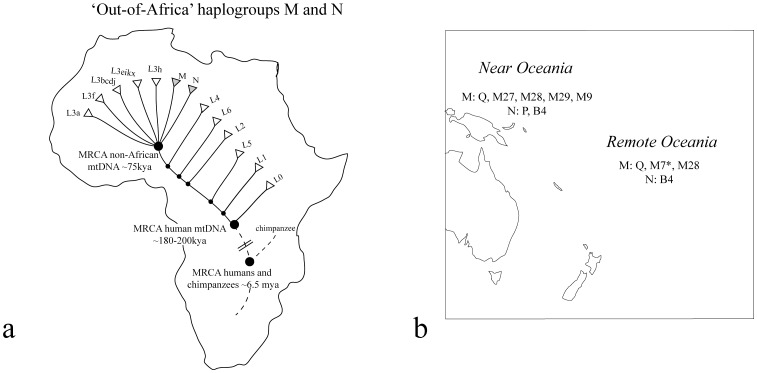
The distribution of mitochondrial haplogroups around the world. 1A. A map of Africa with haplogroups L0–L2 and L4–L6 within Africa. L3 has five lineages within Africa, but the M and N spread out of Africa to the rest of the world, see Behar et al. [21]. 1B. The main mitochondrial lineages found in Near and Remote Oceania (data from whole genome studies).

World-wide, the mitochondrial lineages of humans fit into a strong biogeographic (phylogeographic) pattern [19]–[23]. The oldest lineages (including L0, L1, L2, L4, L5 and L6) are African, and then the L3 subset has a 7-way divergence – with some five L3 lineages still within Africa but also two lineages (M and N) that include the sequences outside Africa (Figure 1A). Thus the L3 group has two deeply diverging mitochondrial lineages (M and N) outside Africa, and both occur within Australia and Oceania. The terminology of haplogroups (lineages) is well defined, but because the terminology evolved (rather than showing forethought and intelligent design!) it can appear somewhat arbitrary. The Q2a lineages reported here are a subset of M, the other major lineages in the region are P and B4 (subsets of N); Figure 1B shows the main haplogroups described from whole mtDNA studies in Oceania.

Genetic studies of the expansion beyond the Solomon Islands into Remote Oceanic islands have identified the B4a1a1a haplogroup as characteristic of the maternal lineage of Polynesians [24], and it is readily traced as far back as the indigenous Formosan people of Taiwan [25]. Further analysis of the genetic variation in mtDNA (mitochondria DNA) and the NRY (non-recombining Y chromosome) in Polynesians by Kayser et al. [12] estimated that about 94% of Polynesian mtDNAs are of East Asian origin (this proportion is higher in the New Zealand Maori population [15]). In contrast to the mitochondrial result, the same studies [12] estimate that 66% of Y chromosome markers (and 21% of autosomal genes) are of Melanesian origin. The genetic results of Wyeth [26] for the Ngai Tahu people of the South Island of New Zealand are in agreement with this, and show (using the population genetics program STRUCTURE) that at least two populations (and quite likely three) contributed to Ngai Tahu genetics, irrespective of the later European contribution. Thus the Ngai Tahu data supports the conclusion that there was an earlier admixture of at least two lineages [12]. From a genetics perspective, higher genetic diversity is desirable (‘hybrid vigour’) and low genetic diversity disadvantageous (‘inbreeding depression’).This observed genetic diversity in Polynesia requires further research, but is certainly consistent with some form of admixture as previously noted [12], [27].

The timing of this Melanesian Y-chromosome influence has not yet been revealed by these analyses; it may have occurred early during the time of the ‘Lapita’ dispersal, or resulted later from ongoing movements of males. Clearly we need more genetic information regarding which mitochondrial lineages occur in Near Oceania in order to help understand why such a small but distinctive subset is found in Remote Oceania. It is important to establish a better understanding of the genetics of populations in Near Oceania, and as a step towards this we report seven new mitochondrial genomes (mtDNA) from the haplogroup Q2a [28]. These were identified from individuals sampled in Harrison [29], [30], and they increase the number of complete mitochondrial genomes within the Q2 haplogroup (subgroup) from four to eleven. These haplotypes were identified in individuals from Papua New Guinea (PNG), Fiji, and Kiribati.

## Materials and Methods

### Samples

A collection of over 4000 blood samples from Papua New Guinea, Fiji and Kiribati was made for a study investigating the molecular epidemiology and evolution of Hepatitis B virus (HBV), including vaccine escape and occult (low-level undetected) infections [29], [30]. The primary purpose of that study was Hepatitis B virus in the Pacific, and as part of this a selection of markers from the mitochondrial genome and the Y chromosome were sequenced and compared in order to investigate any human genetic components that may aid understanding transmission and/or clearance of the virus. In PNG and Fiji, permission was obtained via the PNG National Medical Research Advisory Committee and the Fiji National Research Ethics Review Committee respectively. In Kiribati there was no established ethical review committee at the time of the project, therefore extensive consultation with the Ministry of Health and local medical professionals was conducted before the project began. Further details, including examples of consent forms, are in Harrison [29].

Because of both the high inter-provincial and between country migration throughout Oceania it was important to record the family history of all the volunteers who donated blood samples. The PNG samples were collected in Port Moresby, Hagan, Goroka, Madang and Daru, and include individuals originating from all 19 provinces. PNG is highly diverse culturally, linguistically and genetically [1], [19] and was colonised in the last 40–60,000 years. The two Q2 haplotypes found in Port Moresby were of West New Britain origin. The Fijian samples were collected in Suva from nationals living in the city; modern Fiji is a major regional centre attracting immigrants from throughout Oceania. Historically, Fiji was settled about 3000 ybp by the same founding Lapita population that eventually colonized Tonga. However, Fiji (unlike Tonga and Samoa) continued to receive both genetic and cultural influences from further west that make it distinct from Polynesian countries. The Kiribati samples were collected from North Tabiteua and South Tarawa and include individuals from all 12 atolls of the Gilbert Islands of Kiribati. The I-Kiribati people are suggested [1] to be a mixture of Lapita, and Malay ancestry, and the atolls are suggested to have been colonised from three directions about 2–3 KYA bp: the western archipelagos of South East Asia, the Southern Archipelagos of the Solomons, and the Bismarck Archipelago.

From the 4,000 samples collected a total of 442 samples (269 from PNG, 112 Kiribati and 61 Fijians) were selected for further study based on the reported ethnicity of their maternal grandmother and/or paternal grandfather. Sequencing of the first hypervariable region of the mitochondrial DNA, and typing of the non-recombining part of the Y-chromosome (NRY), was carried out as in Hurles *et al.* [31]; 430 samples were successfully typed.

From these samples, eight were identified as having the Q2 mitochondrial haplotype as described in [28]. They were: FA064 and FA093 from Fiji; K058 and KI018 from Kiribati; and P054, PB036, PD057 and PD047 from Papua New Guinea. P054 was not available for full mitochondrial analysis because the limited sample was reserved for Hepatitis B virus analysis. In addition, for methodological controls we sequenced two other mitochondrial genomes. The maternal ancestral origins for all nine genomes are given in Table 1.

**Table 1:** Locations for ancestral maternal lineages.

Sample	GenBank #	Country	Village	District/Atoll	Province	Latitude/Longitude	Haplogroup
FA064	GQ214521	Fiji	Bukuya	Nadrogo	Ba	17.8S/177.7E	M/Q2a
FA093	GQ214522	Fiji	Raiwasa	Rakiraki	Ra	18.9S/178.3E	M/Q2a
KI018	GQ214525	Kiribati	Otowa	Onotoa		1.8S/175.6E	M/Q2a
K058	GQ214524	Kiribati	Temotu	Nonouti		0.5S/174.2E	M/Q2a
PD047	HQ113226	PNG	Madamee	Daru	Western Province	9.1S/143.2E	M/Q2a
PB036	GQ214526	PNG	Talasea		West New Britain	5.4S/150.0E	M/Q2a
PD057	GQ214527	PNG	Sirra	Manus Is	Manus province	2.1S/147.0E	M/Q2a
K040	GQ214523	Kiribati	Onomaru	Butaritari		3.1N/172.8E	N/R/B4a1a1a1
CAC	GQ214520	Scotland		Sutherland	NW Scotland	58N/4W	N/R/U5

### DNA Extraction, PCR Sequencing and Alignment

Total genomic DNA was extracted from the blood samples using the Wizard® Genomic DNA Purification Kit (Promega) and the High Pure PCR Template Preparation Kit (Roche Applied Science) according to the manufacturer’s protocols. Long range PCR was performed using the Expand Long Template kit from Roche. All PCR products for this study were generated using primers from the Rieder primer set [32]. Long range PCR was performed with primers 1F/11R and 11F/1R and the following protocol: 93°C for 4 mins, 10 cycles of 93°C for 30 secs, 50°C for 30 secs, 68°C for 8 mins, followed by 25 cycles of 93°C for 30 secs, 50°C for 30 secs, 68°C for 8 mins +20 sec per cycle, a final elongation step at 68°C for 20 mins and a hold at 10°C. In some cases it was not possible to generate long fragments from the genomic DNA extraction; in these cases, smaller fragments of 4–6 kb were generated. And if this failed, overlapping 1 kb fragments were generated directly from total genomic DNA.

The rationale for using long fragments as templates to make shorter products suitable for direct sequencing is to avoid amplifying pieces of mtDNA that have been inserted into the nucleus (numts - nuclear copies of mitochondrial sequences) and are therefore evolving differently from the mitochondrial DNA itself (ref 32 takes this into account). During our study, the mitochondrial genome of one of us (CAC) was initially sequenced across the control region using both total genomic DNA and a long fragment as template for the smaller products (23F/R, 24F/R and 1F/R). All sequences were identical and matched perfectly to adjacent sequences which gave us confidence to generate the smaller products directly from the genomic DNA. All short products (1–4 kb) were made in reactions containing: 1XReaction Buffer (ABgene), 1.5 mM MgCl_2_, 250 µM dNTPs, 1U Taq (Red Hot Taq, ABgene), 10 µM each primer and 10–50 ng of template. The programme for the PCR was: an initial denaturation of 94°C for 3 mins followed by 35 cycles of 94°C for 30 secs, 50°C for 30 secs, 72°C for 1–4 mins depending on the size of the product, followed by a single cycle of 72°C for 5 mins and a hold at 10°C.

All genomes but one were sequenced using standard Sanger sequencing methods on an ABI 3730 DNA Analyzer (Applied Biosystems); for the sample FA64, two long-range PCR products were amplified and sequenced on the Illumina GAII platform and assembled using an in-house computer program [33], [34]. Sequences were edited and assembled in Sequencher 4.7™ (Gene Codes Corporation, USA). Consensus sequences for each of the genomes were compared to the revised Cambridge Reference Sequence and to the four other Q2 complete mt sequences from GenBank (AY956412–AY956414 and EF495218). Differences to the revised reference sequence were scored in Sequencher 4.7. We also used the error checking techniques of Salas *et al.* [35].

### Analyses

The seven new Q2 mt genomes were aligned with haplogroup Q sequences from GenBank [28], [36]–[40] in a dataset of 36 complete mitochondrial genome sequences. PAUP* (version 4.0b10) [41] was used to find the most parsimonious trees for the data set by heuristic search, after excluding any gapped characters (branch swapping = Tree Bisection-Reconnection, stepwise addition = simple). Nucleotides nt 550–16,050 were included in the analysis; the control region was excluded because it contains several highly variable sites. The parsimony score of the set of trees returned was evaluated using the MinMax Squeeze programme [42] which takes the parsimony score found by the heuristic search as an upper bound and derives a lower bound by summing the parsimony scores of partitions of the dataset. If the upper and lower bounds meet then for that dataset, and by the theorem of Hendy [43], the most parsimonious trees found by heuristic search are proved to have the minimum possible number of mutations. Thus there are several advantages for the analysis of results [44] and for closely related sequences parsimony is a maximum likelihood estimator [45].

A tree with nucleotide changes identified for the Q2 subtree was reconstructed from the consensus network [46] of the most parsimonious tree set, using lists of variable sites for each sequence generated by Sequencher 4.7™ (Gene Codes Corporation, USA). The control region polymorphisms were added to this tree. Because we had small numbers of very long sequences, rather than many very short sequences, we used the Kivisild et al. [39] method for estimating times of divergence rather than the Cox [47] approach.

## Results

All nine mitochondrial genomes have been submitted to GenBank and are available through the identifiers GQ214520–GQ214527, and HQ113226. The seven new Q2 sequences were all Q2a and were each unique, as is the additional B4a1a1a1 haplotype and the European sequence which is U5a2a (both the U and B4 haplogroups branch from the R macrohaplogroup). The parsimony approach (minimizing the number of changes on the tree) allows both non-binary trees and the internal nodes to represent existing sequences. Given also the advantages of proof of minimality (see later), and as we are close to the zone where maximum parsimony equals maximum likelihood, it was advantageous to use the parsimony approach to allow both non-binary trees and internal points to potentially represent observed sequences.

**Figure 2:**
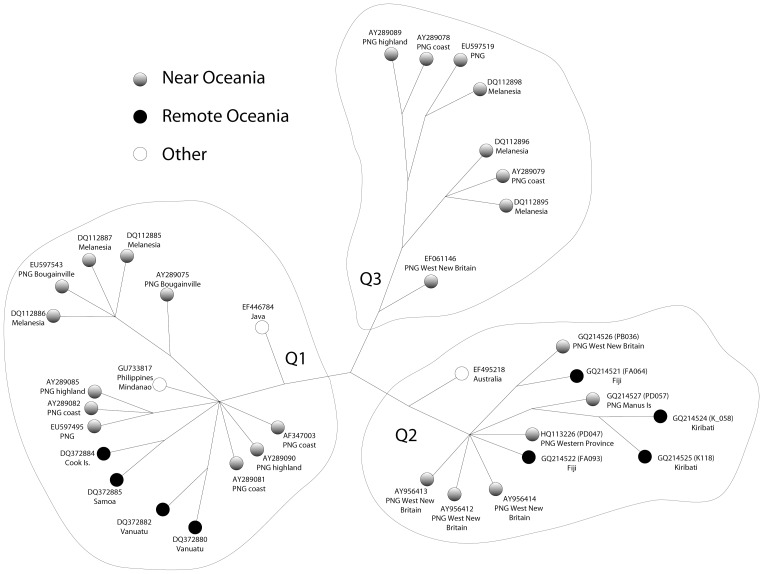
Overview of the Q haplogroup. The dataset has 36 mitochondrial genomes including all eight Q3 sequences, 17 Q1, three Q2 genomes from Friedlaender et al. [28], one from Hudjashov et al. [36], together with the seven additional Q2a genomes reported here. The network has been proved the shortest possible (the minimum number of mutations) by using the techniques in Pierson et al. [40]. Differences in branching between the four equally parsimonious trees occur in the Q3 subgroup.

**Figure 3:**
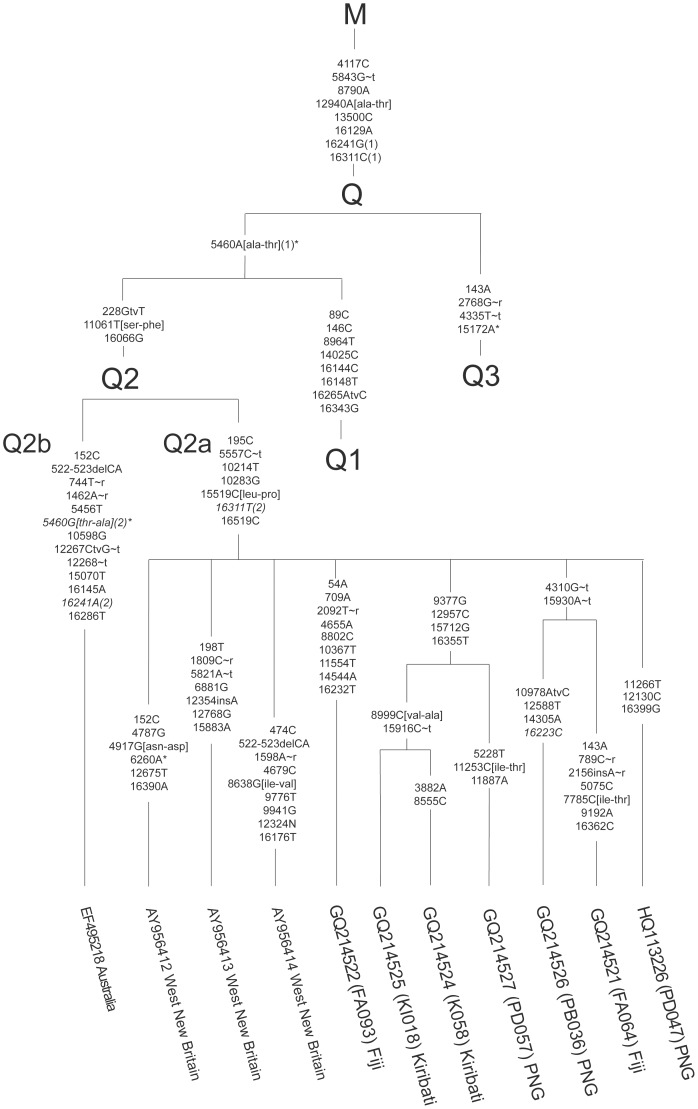
The Q2 haplogroup tree, including seven new Q2a mitochondrial genomes identified in larger type. The figure is a branch-labeled phylogeny of the Q2 haplogroup. Unless otherwise indicated, the base changes shown are transitions from the revised Cambridge Reference Sequence (rCRS, [48]). A transversion is indicated by ‘tv’, for example 228 GtvT is a transversion from guanine to thymine at position 228 on the basal lineage leading to Q2. Changes in RNA genes are shown by suffixes: ‘∼r’ for ribosomal genes, and ‘∼t’ for tRNA genes. Those in italics represent reversions to the same base as the rCRS. Non-synonymous changes within protein-coding genes are described by three-letter amino acid codes following the base number in square brackets. For example, ala-thr represents an amino acid change from alanine to threonine resulting from the substitution at that nucleotide. Deletions are marked by ‘del’. An asterisk indicates the position required more than one step in the parsimony analysis (see Fig. 2). Positions requiring recurrent mutations within the tree have a bracketed number following the description indicating the first and second changes at the site. The changes relative to the rCRS at the M vertex are: 73G, 263G, 489C, 750G, 1438G, 2706G, 4769G, 7028T, 8701G, 8860G, 9540C, 10398G, 10400T, 10873C, 11719A, 12705T, 14766T, 14783C, 15043A, 15301A, 15326G, 16223T.

The seven new Q2a mitochondrial genomes analysed together with the available Q lineages (Q1 & Q3). A heuristic tree search from our alignment of the coding-regions of 36 complete mt genomes, and with 140 informative characters, found six equally parsimonious trees with a tree score of 145, and these are the shortest possible trees for this data. Thus only 5 nucleotides (sites 5460, 6260, 92547, 13368, and 15172) had repetitive (parallel or reverse changes) changes. In this case it is relatively easy to partition the sites to demonstrate that no shorter tree is possible (that is, for this data there is no tree with fewer mutations); this was done using the MinMax Squeeze program [42]. For example, by comparing the results for Figure 2 and 3 we find that the reversion at site 5460 (on the Australian Q2) is separated from the original change by sites 228, 11061 and 16066. In principle, we can express this as a rectangle with 5460 on both short sides, and 228, 11061 and 16066 on the long sides. To fit this rectangle onto any tree (which has no cycles, such as rectangles) we have to remove one side. The choice is, removing a side with a single duplication on 5460 (leaving two branches with three duplications, one each at 228, 11061 and 16066), or removing one side with three duplications (leaving only the site 5460 with a duplication).

Clearly, the second alternative is the simplest (most parsimonious) hypothesis - leaving a single change on 5460, rather than three changes on 228, 11061 and 16066. This general approach is then extended to the other sites where more than one mutation occurs, eventually giving a proof of minimality. Thus there cannot be a shorter tree, even if there could be up to four trees with the same number of mutations. We consider it very important that trees are proven to be of minimal length, the proof of minimality is too often omitted with haplotype data.

There is a potential cycle in Figure 2 at an internal point in Q3– but this does not affect our analysis of Q2. The individual changes along each branch within the Q2 group are also shown on Figure 3. Unless otherwise indicated, the base changes shown are transitions from the revised Cambridge Reference Sequence (rCRS) [48]. It is very interesting that there is a 7-way polytomy at the base of the Q2a sequences. This probably implies a relatively rapid population expansion sometime after Q2a arose (leading to the 7-way division), perhaps similar to the expansion inferred in ref [49]. However, because each extant Q2a sequence is quite different, it implies that this initial population expansion has been followed by relatively distinct populations of Q2as (leading to long isolated lineages). The large number of Q2a lineages would tend to be maintained if there was limited movement of females. Very possibly, the main Q2a group arose about the time of settlement of New Britain (the apparent center of diversity of Q2a), but more information is required before this could be considered a reasonably established hypothesis. In addition, the seven new sequences fall into four groups: two sequences (FA093 and PD047) descend immediately from the unresolved Q2a vertex. Three sequences (PD057, K058 and KI018) descend from a sub-branch from the Q2a vertex defined by three coding-region transitions plus a control region transition. The third set of two sequences, PB036 and FA064, form a second sub-branch from Q2a marked by two shared coding-region transitions.

Using the Kivisild et al. [39] dating method (synonymous amino acid changes only, with the average rate of 1 change per 6760 years, as described in that paper) the estimate for the time to the most recent common ancestor (TMRCA) of the Q2 haplogroup is 34,650±4410 years. The TMRCA estimate for just the Q2a haplogroup is 22,430±3830 years. The three sequences which show some structure within Q2a (K058, KI018 and PD057) have an estimated MRCA at 6760±3050 years. The FA064 and PB036 samples have shared changes after the Q2a vertex but these will not be reflected in the Kivisild MRCA test (both mutations occur in transfer RNAs). These estimates are consistent with those of Friedlaender et al. [28]. We also checked the divergence estimates with the Soares’ method [50] using the calculator provided and their complete sequence rate for all mutations excluding those at positions 16182, 16183, 16194 and 16519. We obtained almost identical results. Both Kivisild et al. [39] and Soares et al. [50] were concerned about the ‘apparent rate acceleration’ – the J-shaped curve – that could have affected more recent time estimates. However, we have recently shown [51] that that issue is not a problem here. The question only arises when the existing genetic diversity in a population is not accounted for; in contrast, it is not an issue when given the full phylogenetic analysis. These dating estimates are within the time that modern humans have lived in Near Oceania; thus it is likely (given also that Q2 has not been found outside Near Oceania and Australia) that the Q2 lineage arose in the region, and is not a later intrusion (see, for example, [18]). The final aspect of these Q2 results is that there is some regional specificity in Near Oceania, which will put constraints on the understanding of population numbers, in this case of females.

The new B4a1a1a1 haplotype (K040, GQ214523, Table 1) is also a novel sequence that has not been reported previously, though the haplogroup is relatively common within Kiribati (60 in our samples). This K040 sequence has the full Polynesian motif (see Figure 3 of ref [40]). The sole European mt genome reported here is interesting in that it is a deeply diverging U5a (or more fully, N/R/U5a; see [52], [53]). In general, the different U5’s are quite widely spread across Europe (with some in North Africa) and are suggested to predate the spread of agriculture into Europe. However, this distinctive version has a maternal ancestry from North-West Scotland (Table 1).

## Discussion

Our results mean that Q2a is now better represented by complete mitochondrial genomes. In order to identify and to eventually model and understand more about earlier populations it is necessary to build up knowledge of the overall genetic diversity and frequencies of existing mitochondrial and Y-chromosome haplogroups. As noted in the Results, it is interesting that the increase in the number of complete mitochondrial genomes of the Q2 lineage does not significantly affect estimates of timing for the origin of the lineage [39], [50]; the estimates appear stable in that regard.

Overall, this leads to our primary result that the Q2 haplogroup, and probably the ancestral Q as well, most likely arose from the M haplogroup within the general region of Near Oceania. It is likely that the Q lineage itself arose soon after the time of human arrival in Australia and New Guinea region [54]. These authors report that in parts of Melanesia there is high regional genetic specificity, and the difference between inland and coastal populations can be quite large. This implies long-term relative stability of some populations, and our finding of the relatively localised distribution of the Q2a haplogroup is in agreement with those findings. In general, we need to see agreement between datasets, but it may be a while before we have sufficient data to address this more quantitatively [44].

One of our longer term goals is to be able to model the founding population size of early humans in the region, similar to that which has been done with Aotearoa New Zealand [15]–[17]. The short term goal is to get more data on the diversity and frequencies of existing mitochondrial genomes. Despite the Q haplogroup being relatively common in Near Oceania (Melanesia), the Q2 haplogroup itself is not common, occurring in only 2% of our Oceanic samples and in 9.6% of Friedlaender’s Island Melanesia samples. Interestingly, they [28] reported Q2a in about 15% of samples from New Britain, so this may well be its center of distribution. However, its distribution is not really part of this study – we are basically considering the deep lineages, not yet their frequencies and geographic distribution. This certainly does not preclude the Q2 haplogroup being regionally abundant elsewhere, but because Q2 is both relatively old and diverse, it emphasizes the long period that humans have been in the region. Nevertheless, despite being old, it is reasonably localised – compared with the distribution of haplogroups of equivalent age in, say, Europe.

Of note is that only Q2 (not Q1 or Q3) was found in our Fiji and Kiribati samples, giving support to a reasonably high regional specificity in the maternal lineages. This was unexpected in a total of 430 samples typed, (and which came from PNG, Kiribati and Fiji) given previous reports of Q1 in Fiji, the Cooks and Samoa. The Kiribati Q2a sequences cluster together – possibly implying a common source from a limited region, though the time to their common ancestor could still be several thousand years ago. However, neither the PNG nor the Fiji sequences cluster, possibly suggesting that the Fiji ancestors come from different regions of Near Oceania? The result from Fiji and Kiribati emphasizes that while a wide range of mitochondrial haplogroups are currently found in Near Oceania the diversity reduces considerably as the people move eastward. Given only the maternal element, it could have been assumed that Polynesians had limited genetic diversity. This, as mentioned earlier, does not seem to be the case, there is significant genetic variability in nuclear DNA in Polynesians, as measured among Ngai Tahu people in southern New Zealand [26], [55]. However, given the relatively low mitochondrial diversity among Polynesians, this means that that the additional variability may come from an influx of males.

At present we cannot determine when the additional paternal genetic diversity moved into Remote Oceania. There is a range of possibilities which we could perhaps represent as a spectrum. At one end of the spectrum, the additional paternal diversity could have been established at (or before) the ‘Lapita expansion’ (as defined by the archeological record [1]). At the other end of the spectrum, it could have begun after the initial settlement of Polynesia with an ongoing influx (especially of males) from Near Oceania into Remote Oceania. Reality may be somewhere in the middle, but there is little data yet to establish a preferred position. We note that some oral histories in Fiji [56] refer to people coming into Fiji from ‘the West’, and marrying local women; as such this could be part of an ongoing influx of males. Here, input from a range of social sciences is necessary to establish whether such practices were common [57] and to help model the population history. The first option (full diversity from time of the Lapita expansion) would need to explain the decrease in genetic diversity in going from Melanesia to Western, and then to Eastern Polynesia. A range of alternative hypotheses were presented in Murray-McIntosh et al. [15], and included options with genetic and/or social selection. Needless to say, we would also like to see more focus on the independent testing of the components of the models [2].

It is necessary to keep integrating across all forms of data, and it is axiomatic that more data will be required, and the need to integrate across many sources of data is the conclusion of a recent review [58]. One obvious source of data is ancient DNA (aDNA) studies from early human remains, but in all aspects of data collection, critical new information will almost certainly become available [59]. Perhaps a positive effect from the new results over the last decade (including the present results) is that they help highlight areas where additional information will be critical; the extent of matrilinearity in early (pre-missionary) societies [7], [56], [60] is just one such example where further information is required in order to model the genetics more accurately.
